# Imerslund-Gräsbeck syndrome in a child with a novel compound heterozygous mutations in the *AMN* gene: a case report

**DOI:** 10.1186/s13052-024-01757-z

**Published:** 2024-09-27

**Authors:** Dedong Zhang, Siying Liu, Bixin Xi, Yongbing Zhu, Yu Chen, Jiasi Zhang, Aiguo Liu

**Affiliations:** Department of Pediatrics, Tongji Hospital, Tongji Medical College, Huazhong University of Science and Technology, Wuhan, 430030 China

**Keywords:** Imerslund-Gräsbeck syndrome, *AMN* gene, Anemia, Oral vitamin B12, Case report

## Abstract

**Background:**

Imerslund-Gräsbeck syndrome (IGS) is an autosomal recessive disorder characterized by selective vitamin B12 malabsorption, resulting in vitamin B12 deficiency and impaired reabsorption of proximal tubular proteins.This case highlights a previously unidentified compound heterozygous variant in the Amnionless (*AMN*) gene that causes IGS syndrome and underscores the importance of long-term oral vitamin B12 replacement therapy in managing the condition.

**Case presentation:**

In this retrospective analysis, we present the clinical data of a 3-year and 6-month-old female child diagnosed with IGS at Tongji Hospital, Tongji Medical College, Huazhong University of Science and Technology, China, in November 2018. The child was admitted to the hospital due to a history of anemia persisting for over a month. There was no previous significant medical history. The admission examination revealed megaloblastic anemia with proteinuria. Serum vitamin B12 levels were decreased, while folic acid and renal function were normal. The patient was diagnosed with megaloblastic anemia and started long-term oral vitamin B12 replacement therapy. Throughout the follow-up period, blood tests consistently showed normal results, while proteinuria persisted. In November 2019, the child and her parents underwent whole exome sequencing analysis, which revealed a novel compound heterozygous variant in the *AMN* gene: c.162 + 1G > A and c.922 C > T (p.Q308X) in the child, c.162 + 1G > A in the father, and c.922 C > T (p.Q308X) in the mother. Therefore, this child was further diagnosed with IGS.

**Conclusions:**

In this case, whole exome sequencing proves to be highly practical in daily healthcare for diagnosing and refining rare or ultra-rare diseases with ambiguous phenotypes or genetic diversity. It is also valuable for prognostic evaluation and personalized management. Additionally, the oral vitamin B12 treatment demonstrated positive clinical effects for the child, offering a new option for patients unable to undergo intramuscular vitamin B12 replacement therapy.

## Background

Imerslund-Gräsbeck syndrome (IGS) is characterized by megaloblastic anemia in infants and young children, with reduced serum vitamin B12 levels and mild small molecular proteinuria. It can present with secondary symptoms related to vitamin B12 deficiency, such as stunted growth, loss of appetite, fatigue, lethargy, or recurrent infections [1]. The first report of IGS was by Olga Imerslund and Ralph Gräsbeck in 1960, and most cases have been documented in Finland, Norway, and Eastern Mediterranean countries [2]. This case presents a newly identified compound heterozygous variant in the *AMN* gene, consisting of a novel exon variant c.922 C > T (p.Q308X) and a novel intron variant c.162 + 1G > A. The present patient diagnosis was made by whole exome sequencing, and favorably treated with vitamin B12 supplementation.

## Case presentation

### General information

The child, who was 3 years and 6 months old, was admitted to the hospital due to persistent anemia for over a month. The child was delivered naturally at full term, and their personal history indicated a good nutritional balance with no selective eating habits. However, there was a recent episode of consuming broad beans and a tendency to wake up easily during sleep. There were no significant past medical or family history, and the living environment was considered favorable.

### Physical examination on admission

The patient presented with a body temperature of 38.3℃, a pulse rate of 122 beats per minute, a respiratory rate of 30 breaths per minute Her weight was 14.6 kg. According to the World Health Organization growth standards, it was at the 45th percentile. Physical examination revealed a pale complexion and lips, with no visible rash or bleeding spots on the skin or mucosa. There was no enlargement of superficial lymph nodes and no congestion in the pharynx. Bilateral tonsils showed grade I enlargement. The neck felt soft, while coarse breath sounds were detected in both lungs. No dry or wet rales were heard from her lung. Heart sounds were strong and regular, with no apparent pathological murmurs in any valve area. The abdomen was soft, and no abnormalities were noted in the liver and spleen beneath the ribs. The nervous system appeared normal without any noticeable abnormalities.

### Laboratory results

The hematological parameters indicated macrocytic anemia and reduced serum Cobalamin level (vitamin B12 < 50 pg/mL)in the presence of normal serum folate levels (20.54ng/ml), hemoglobin content was 61 g/L (118.0-156.0g/L), mean corpuscular volume 112.7fL (118.0-156.0g/L), mean hemoglobin content 38.6pg (25.0-34.0pg), reticulocyte 2.32% (0.50-1.50%), free hemoglobin < 25 mg/L (<40.0mg/L). Lactate dehydrogenase was 1116 U/L (120-300U/L), the creatinine was 26µmol/L (45-84µmol/L); Ferritin was 177.8 µg/L (15-150µg/L), the serum iron was 28.27µmol/L (6.6-26.0µmol/L), the unsaturated iron binding capacity was 14.5µmol/L (24.2-70.1µmol/L); urinary protein was ‘++’; Erythrocyte fragility test, erythrocyte cluster of differentiation 59, and thalassemia genes were normal, hemoglobin electrophoresis showed elevated hemoglobin F; peripheral blood smear and bone marrow smear showed megaloblastic anemia; urine test results were shown as follows, α1-microglobulin was 9.20 mg/L (0-5mg/L), urinary microprotein was 534 mg/L (≤150.0mg/L), urinary microalbumin was 323 mg/L (≤20.0mg/L), urinary protein electrophoresis showed that urinary microalbumin accounted for 89.7% and transferrin accounted for 10.3%. 24 h urinary microalbuminuria total protein 366.1 mg/24 h (≤140.0mg/24h), 24 h urinary microalbuminuria 234.1 mg/24 h (≤30.0mg/24h); liver, gallbladder, pancreas, spleen, kidney and heart color ultrasound showed no abnormality.

### Genetic test results

**Fig. 1:**
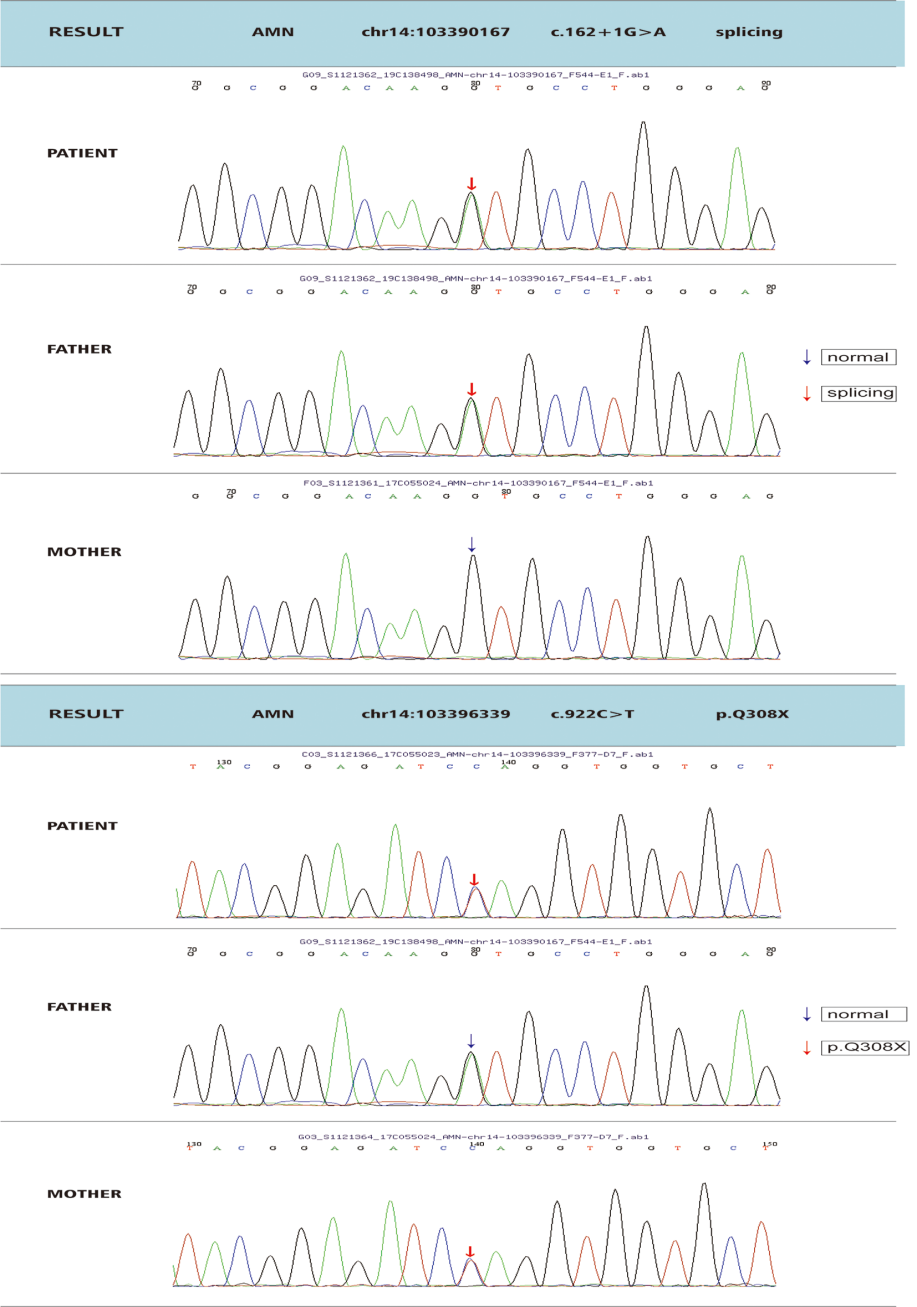
Genetic test results and analysis of children and their parents. The results of *AMN* gene analysis of the child and his parents showed that the child and his father carried *AMN*: c. 162 + 1G > A heterozygous variant, the child and her mother carried *AMN*: c. 922 C > T heterozygous variant

**Fig. 2:**
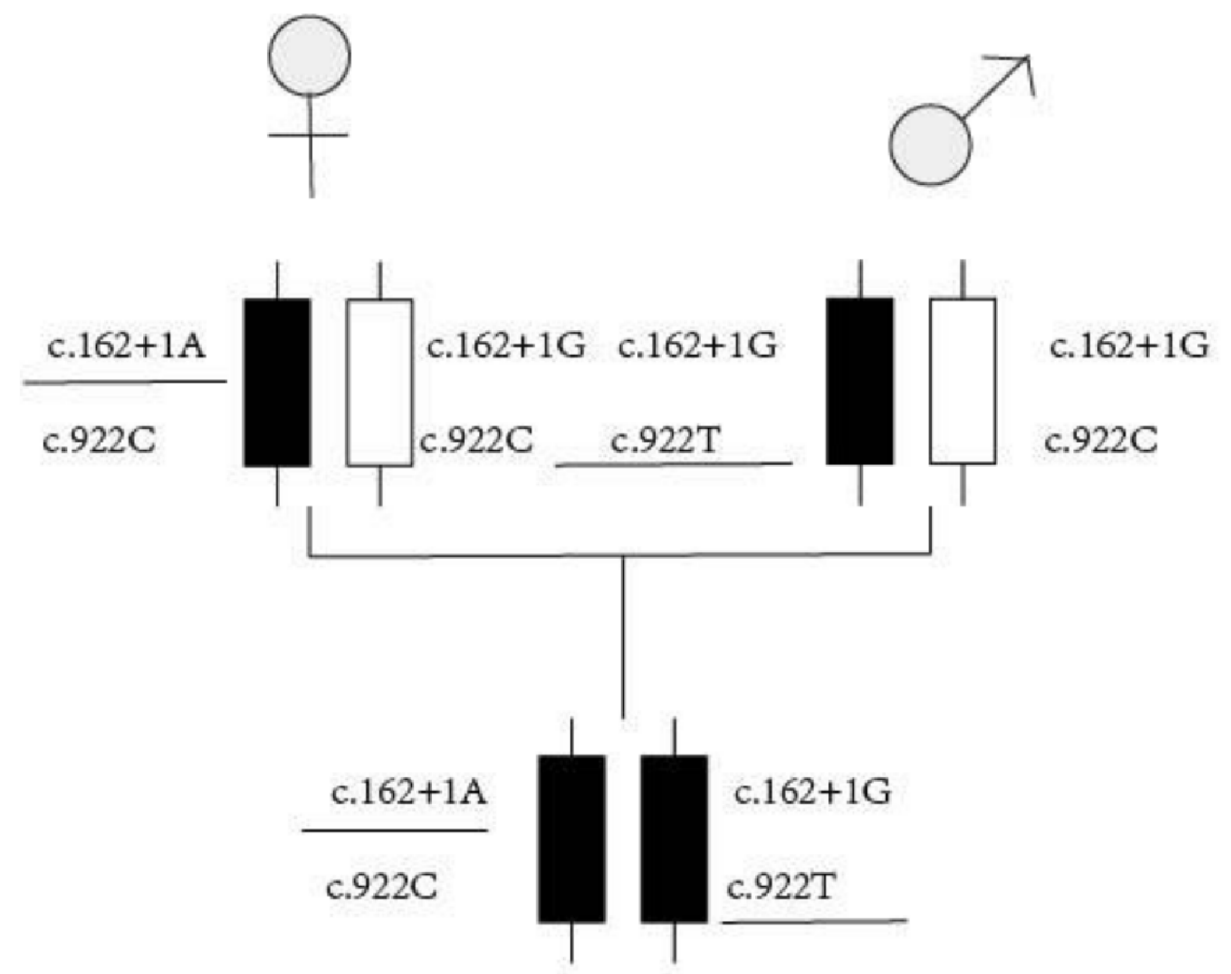
pedigree analysis diagram

Based on the high-throughput sequencing results, the child in question has been found to have a compound heterozygous variant in the *AMN* gene: c.162 + 1G > A and c.922 C > T (p.Q308X). Further analysis of the parents’ genetic information revealed that the father carries the c.162 + 1G > A heterozygous variation, while the mother carries the c.922 C > T (p.Q308X) heterozygous variation (Fig. 1).In accordance with the guidelines provided by the American College of Medical Genetics and Genomics, the c.162 + 1G > A mutation (exon 2, NM_030943) is classified as a splicing mutation that can potentially result in an amino acid change and loss of protein function. This mutation has been preliminarily determined to be pathogenic. Furthermore, pedigree analysis indicates that the mutation is present in a heterozygous state in the father but not in the mother, consistent with the autosomal recessive inheritance pattern observed in Inherited Glomerulopathy Spectrum. As for the c.922 C > T mutation (exon 9, NM_030943), it is classified as a nonsense mutation, leading to the loss of amino acid change and loss of protein function. Through pedigree verification analysis, it was found that the locus is not mutated in the subject’s father, while the subject’s mother carries a heterozygous mutation at this locus. This pattern aligns with the pathogenic autosomal recessive inheritance pattern observed in IGS (Fig. 2).

### Diagnosis and treatment and follow-up

After the initial diagnosis of macrocytic anemia, the child received oral treatment with vitamin B12 at a dosage of 100 µg/day. After 3 months of treatment, the outpatient follow-up revealed that the hemoglobin levels had returned to normal. However, due to the persistent proteinuria, a whole exon gene test was conducted in November 2019, which led to the diagnosis of IGS.Despite the diagnosis of IGS, the child continued to receive oral vitamin B12 therapy as an alternative treatment during follow-up. Fortunately, the oral vitamin B12 therapy demonstrated good efficacy for the child. As a result, the decision was made to maintain the adherence to oral vitamin B12 therapy even after the diagnosis of IGS. The results of hemoglobin, vitamin B12, folic acid, urinary protein and renal function during the follow-up period were as follows (Table 1).

**Table 1:** Examination results of children during diagnosis and treatment

Stages	Hemoglobin (g/L)	Vitamin B12 (pg/mL)	Folic acid (ng/mL)	proteinuria	serum creatinine (umol/L)
2018.11	61.0	<50	20.54	++	26
2019.1	115	104	19.85	*	*
2019.6	131	158	18.31	*	*
2019.12	122	262	11.28	*	*
2020.8	121	145	12.71	++	29
2021.8	135	520	15.67	++	28
2022.1	105	207	14.71	++	42
2023.8	122	297	10.84	++	35

*: data missing

## Discussion and conclusions

IGS was caused by mutations in either of the coding Cubilin (*CUBN*)or Amnionless genes with loci of 10p12.1 and 14q32, respectively. Cubilin is a multifunctional receptor weighing 456 kDa that lacks transmembrane and cytoplasmic domains. It is predominantly expressed in the apical brush border membrane of polarized epithelial cells. On the other hand, Amnionless is a transmembrane protein with a molecular weight of 50 kDa [2], enabling the Cubilin protein to function by tightly binding to the N-terminus of Cubilin.Together, cubilin and amnionless form a receptor complex known as cubam. The cubam complex acts as an intrinsic factor-vitamin B12 receptor, facilitating the uptake and transport of vitamin B12 in various tissues and cells [3]. Cubam exhibits high expression levels in the small intestine and proximal renal tubules, where it performs crucial functions. It plays a significant role in the uptake of vitamin B12 in the intestines, facilitating its absorption. Additionally, Cubam is involved in the reabsorption of proteins in the epithelial cells of the renal proximal convoluted tubule. Furthermore, it plays an important role in early embryogenesis, contributing to the development and growth of embryos during the initial stages [1, 4]. As of January 2023, the Human Gene Mutation Database [5] has documented a total of 60 different mutations in the *CUBN* gene and 35 different mutations in the *AMN* gene. Among the 35 *AMN* gene mutations, 9 were identified as nonsense mutations, 9 as splicing mutations, 8 as microdeletion mutations, 4 as microinsertion mutations, 1 as a microdeletion and insertion mutation, and 4 as fragment deletion mutations. These mutations have been cataloged and studied for their potential impact on the respective genes. Since the first case of IGS with a homozygous mutation in the *AMN* gene locus was reported at Shenzhen Children’s Hospital in China in 2019, there have been a total of 7 reports documenting 8 cases of IGS with mutations in the *AMN* gene locus in China(Table 2). The comprehensive gene detection conducted on the child (Fig. 1) revealed a new compound heterozygous pathogenic variation in the *AMN* gene: c.162 + 1G > A and c.922 C > T (p.Q308X).According to the ACMG guidelines, the c.162 + 1G > A mutation in exon 2 of the *AMN* gene (NM030943) is classified as a splicing mutation. This mutation can lead to the appearance of a premature stop codon or exon skipping, which can trigger nonsense-mediated mRNA degradation or alter the normal protein structure through exon skipping. Therefore, this mutation is considered a pathogenic variant. The c.922 C > T mutation in exon 9 of the *AMN* gene (NM030943) is classified as a nonsense mutation. It replaces the amino acid glutamine with a premature stop codon at position 308, which can result in the truncation of the protein. This mutation can trigger nonsense-mediated mRNA degradation, leading to the degradation of mutated mRNA, or affect protein function by altering the conformation of the protein in the beta-sheet region where the 308th amino acid is located. Consequently, this mutation is also classified as a pathogenic variant.

Unlike hereditary intrinsic factor deficiency, patients with IGS do not typically exhibit symptoms immediately after birth. The age at which symptoms manifest can vary, ranging from a few months to 14 years [6].In this specific case report, the patient presented with symptoms at 3 years and 6 months of age, which aligns with the reported age of onset in the available literature. The clinical presentation included features such as megaloblastic anemia, serum vitamin B12 deficiency, and persistent mild proteinuria. These signs were indicative of IGS and further supported the suspicion of the condition. The diagnosis of IGS was ultimately confirmed through whole exome sequencing analysis conducted one year later. Due to vitamin B12 deficiency, the child quickly received replacement therapy with vitamin B12.Interestingly, in this case, oral vitamin B12 treatment also exhibited positive clinical effects. The child’s anemia and symptoms associated with vitamin B12 deficiency showed significant improvement during outpatient follow-up. These findings align with a study conducted by Gangarossa et al. in 1996 [7] and a study conducted by Neven et al. in 2023 [8], which likely reported similar positive outcomes with oral vitamin B12 supplementation. Based on the research conducted using databases such as PubMed, HowNet, and Wanfang, it is evident that most related case reports on the replacement treatment of vitamin B12 primarily utilize intramuscular injection [1, 6, 9–20].Based on the pathogenesis of IGS, where selective malabsorption of vitamin B12 occurs, the therapeutic efficacy of oral vitamin B12 supplementation may be compromised. However, oral administration of vitamin B12 in this child yielded favorable therapeutic outcomes. Epigenetic modifiers may alleviate selective malabsorption of vitamin B12, which may play a role in it [21]. An earlier trial of vitamin B12 absorption in 30 IGS patients demonstrated a greater heterogeneity in the absorption impairment of vitamin B12 in different IGS patients [22].This heterogeneity may be attributed to differences in the number and activity of vitamin B12-internal factor complex receptors among individuals with IGS. Additionally, molecular changes in the *AMN* genes of IGS patients can result in varying degrees of functional impairment of the vitamin B12-internal factor complex receptor. The *AMN* protein is a transmembrane protein with signal sequence and transmembrane domain located between amino acids 1 and 19 and between amino acids 361 and 381, respectively [23]. The compound heterozygous mutation of *AMN* in this child is located at amino acid 308 (c.922 C > T) and intron 3 (c.162 + 1G > A) of the coding region, which does not affect the key functional domains of the protein. Therefore, the degree of receptor defect caused by *AMN* mutation in this child may be mild. On the other hand, because approximately 1% of oral vitamin B12 is absorbed through passive diffusion in its free form, high doses can effectively treat vitamin B12 deficiency, as the patient of this case [24] .In addition, previous studies have shown that the absorption capacity of vitamin B12 declines from childhood to adulthood, and this patient is young, so the ability to absorb vitamin B12 may be stronger [25]. Finally, the patient followed medical advice and improved her dietary structure by consuming more vitamin B12-rich foods such as beef liver, goose liver, and seafood like octopus and caviar. This also contributed to the improvement of serum vitamin B12 levels [26, 27].

The expression of Cubam in the brush border membrane of intestinal mucosal and renal tubular epithelial cells relies not only on the proper binding of Cubilin and Amnionless but also on the specific N-glycosylation of Cubilin. N-glycosylation is an important post-translational modification that regulates the folding, stability, intercellular transport of Cubilin and Amnionless proteins, as well as the formation of the Cubam complex. The patient exhibits persistent mild proteinuria that does not improve with serum vitamin B12 levels, suggesting that the two mutations in *AMN* likely affect the specific N-glycosylation of Cubilin, thereby impacting the maturation of the Cubam complex and their surface localization [28].

**Table 2:** Treatment method and mutation site of IGS syndrome patients with *AMN* gene mutants

Numbering	Sex	Age	Mode of administration	mutation site	References
1	Female	18 months	intramuscular injection	c.208–2 A > G	[1]
2	Female	24 months	intramuscular injection	c.513 + 5G > A c.1006 + 34_1007-31del	[2]
3	Female	36 months	intramuscular injection	c.513 + 5G > A c.1006 + 34_1007-31del	[2]
4	Female	11 months	intramuscular injection	c.513 + 5G > A c.1006 + 34_1007-31del	[3]
5	Male	8 months	Oral	c.208–2 A > G	[8]
6	Female	4 years	Oral	c.208–2 A > G	[8]
7	Female	36 months	intramuscular injection	c.1225_1226del	[9]
8	Female	18 months	intramuscular injection	c.1225_1226del	[9]
9	Male	12 months	intramuscular injection	c.1225_1226del	[9]
10	Male	39 months	intramuscular injection	c.663G > A	[10]
11	Male	24 months	-	c.208–2 A > G	[11]
12	Female	24 months	-	c.742 C > T	[11]
13	Female	25 months	intramuscular injection	c.208–2 A > G	[12]
14	Male	15 months	intramuscular injection	c.280–2 A > G	[13]
15	Male	8 months	intramuscular injection	c.280–2 A > G	[13]
16	Female	11 months	intramuscular injection	c.280–2 A > G	[13]
17	Male	8years and 8 months	intramuscular injection	c. 43 + 5G > A c.C717G: p.Cys239Tyr	[14]
18	Male	6 years and 2 months	intramuscular injection	c. 43 + 5G > A c.C717G: p.Cys239Tyr	[14]
19	Female	4 years and 7 months	intramuscular injection	c.742 C > T(p.Q248X)	[15]
20	Female	24 months	intramuscular injection	c.742 C > T(p.Q248X)	[15]
21	Female	6 years and 8 months	intramuscular injection	c.742 C > T(p.Q248X)	[16]
22	Male	16 years and 5 months	intramuscular injection	c.742 C > T(p.Q248X) c.761G > A(p.G254E)	[17]
23	Male	10 years	intramuscular injection	c.527_530del c. 651 + 1G > A	[18]
24	Male	3 years and 11 months	intramuscular injection	c.742 C > T(p.Q248X)	[19]

Two novel mutations in the *AMN* gene were identified: c.162 + 1G > A and c.922 C > T (p.Q308X). Oral administration of vitamin B12 during regular replacement therapy has been shown to significantly improve anemia and related symptoms caused by vitamin B12 deficiency in IGS. In cases of megaloblastic anemia, although the increasing rate of families following selective dietary regimens for ideological reasons rather than poverty as in the past in high-income countries, clinicians must be suspicious of rare conditions such as those described here, especially when associated with renal abnormalities [29, 30]. Whole exome sequencing can assist clinicians in diagnostic definition and potentially avoid invasive investigations such as renal biopsy. It can also provide more precise prognostic evaluations, including possible genotype-phenotype correlations, which may inform treatment options [31, 32].

## Data Availability

The datasets used during the current study are available from the corresponding author on reasonable request.

## Abbreviations

*AMN*: Amnionless
IGS: Imerslund-Gräsbeck syndrome
*CUBN*: Cubilin
